# “Near Miss” Obstetric Events and Maternal Deaths in a Tertiary Care Hospital: An Audit

**DOI:** 10.1155/2013/393758

**Published:** 2013-06-26

**Authors:** Roopa PS, Shailja Verma, Lavanya Rai, Pratap Kumar, Murlidhar V. Pai, Jyothi Shetty

**Affiliations:** Department of Obstetrics and Gynecology, Kasturba Hospital, Manipal University, Manipal, Karnataka 576104, India

## Abstract

*Objectives*. (1) To determine the frequency of maternal near miss, maternal near miss incidence ratio (MNMR), maternal near miss to mortality ratio and mortality index. (2) To compare the nature of near miss events with that of maternal mortality. (3) To see the trend of near miss events. *Design*. Audit. *Setting*. Kasturba Hospital, Manipal University, Manipal, India. *Population*. Near miss cases & maternal deaths. *Methods*. Cases were defined based on WHO criteria 2009. *Main Outcome Measures*. Severe acute maternal morbidity and maternal deaths. *Results*. There were 7390 deliveries and 131 “near miss” cases during the study period. The Maternal near miss incidence ratio was 17.8/1000 live births, maternal near miss to mortality ratio was 5.6 : 1, and mortality index was 14.9%. A total of 126 cases were referred, while 5 cases were booked at our hospital. Hemorrhage was the leading cause (44.2%), followed by hypertensive disorders (23.6%) and sepsis (16.3%). Maternal mortality ratio (MMR) was 313/100000 live births. *Conclusion*. Hemorrhage and hypertensive disorders are the leading causes of near miss events. New-onset viral infections have emerged as the leading cause of maternal mortality. As near miss analysis indicates the quality of health care, it is worth presenting in national indices.

## 1. Introduction

As we move closer to 2015, the goal number 5 of the millennium development goals (MDG), to improve maternal health is falling way below our target. Our target in reducing maternal mortality by 75% has not been met with [1]. Pregnant women's health status is not reflected by mortality indicators alone. Hence the concept of *severe acute maternal morbidity* (SAMM) is apt for the present health providing system [2, 3].

SAMM has been studied extensively in the recent past as a complement for maternal mortality and also to evaluate the quality of obstetric care in that particular institution. This concept is superior over maternal death in drawing attention to surviving women's reproductive health and lives and is equally applicable in developing countries as well as developed countries. In many developed countries, maternal mortality has fallen to single digits whereas near miss cases are more and hence useful in evaluation of the present system. Moreover, they have the advantage of not being as rare as maternal deaths for providing adequate information, as well as still being rare enough not to overload clinicians and data collection personnel within the facility [4]. 

Till recently there were no set criteria for identification of these cases for routine implementation, and wider application of this concept was limited [5]. But in 2009, WHO has come up with clinical, laboratory, and management criteria for the identification of these cases [6]. 

Maternal near miss case is defined as “a woman who nearly died but survived a complication that occurred during pregnancy, childbirth, or within 42 days of termination of pregnancy” [6]. 

In our study, we aimed to determine the frequency of maternal near miss, MNM incidence ratio (MNRM), maternal near miss to mortality ratio, and mortality index. Our second objective was to analyze the nature of near miss events and compare the causes of near miss cases with that of maternal mortality. We also saw the trend of near miss events and maternal deaths in two years.

## 2. Material and Methods

An audit of maternal near miss cases from January 2011 to December 2012 was undertaken. Ours is a tertiary care institution with six primary health centers attached to it. It is a referral hospital for both public and private hospitals in Udupi and three other surrounding districts in Karnataka. In addition to providing twenty-four-hour emergency obstetric services, the hospital also provides antenatal care and delivery services for both low and high risk pregnant women. Hospital has 24-hour facility for blood component therapy. High dependency unit (HDU) in labor room complex and intensive care ICU with 24-hour facility for multidisciplinary specialty also function well. 

Potentially life threatening conditions were diagnosed, and those cases which met WHO 2009 criteria for near miss were selected. WHO criteria included a set of clinical, laboratory, and management-based criteria. Maternal mortality during the same period was also analyzed. Patient characteristics including age, parity, gestational age at admission, booked [7] (more than three antenatal visits to our hospital irrespective of the gestational age), mode of delivery, ICU admission, duration of ICU stay, total hospital duration, and surgical intervention to save the life of mother were considered. Patients were categorized by final diagnosis with respect to hemorrhage, hypertension, sepsis, dystocia (direct causes). Anemia, thrombocytopenia, and other medical disorders were considered as indirect causes contributing to maternal near miss and deaths. 

The following near miss indices were calculated. (1) MNM incidence ratio refers to the number of maternal near miss cases per 1,000 live births (LB). MNM IR = MNM/LB. (2) Maternal near miss: mortality ratio: Proportion between maternal near miss cases and maternal deaths. Higher ratio indicates better care. MNM: 1MD. (3) Mortality index: Number of maternal deaths divided by the number of women with life threatening conditions, expressed as a percentage. *The higher the index, is more women with the life threatening condition die* (*low quality of care*), while low index suggests better quality of health care. (MI = MD/(MNM + MD) × 100.

## 3. Results

 During the period of audit there were a total of 7390 deliveries and 7330 live births, 131 near miss cases, and 23 maternal deaths.  Table 1 shows the characteristics of women with near miss and mortality.

 Primiparas were slightly more in the near miss group. Majority of the patients (57.2%) were in third trimester at a near miss event, whereas, in the maternal death group, the number of postnatal patients with puerperal sepsis following section was high (2012). A huge burden of maternal near miss cases 96.2% and 86.96% of maternal deaths were referred.

A total of 755 potentially life threatening conditions were identified of which 131 were near miss cases. Maternal near miss incidence ratio is 17.8/1000 live births. Maternal near miss to mortality ratio is 5.6 : 1. The mortality index is 14.9%. A total of 62.6% of the cases required ICU admission. Among the causes of near miss events, hemorrhage was the leading cause with 44.2%, and hypertension was 23.6%. Third among the group was sepsis, and the last was cardiac disease. 

Sepsis was the leading cause of maternal mortality at our setup, followed by hemorrhage, cardiac disease, and hypertension. We had 5 cases of sepsis in the year 2011, and 7 in 2012. In 2011 we had 3 cases of H1N1 infection with ARDS, one patient with viral pneumonia and other with pyelonephritis. In 2012 there were five cases after cesarean delivery with sepsis, one H1N1 and hepatitis E infection. This accounted for the high mortality due to sepsis.

Table 2 shows the near miss cases and the maternal mortalities. The near miss events for each disorder are given, and mortality index of each condition is also elaborated. It is important to note here that the mortality index for cardiac disease is the highest.

## 4. Discussion

Obstetric deaths represent the quality of maternal care. But for the present scenario it may not reflect the global situation with regard to obstetric care. Hence new “near miss” criteria take over maternal mortality ratio. Although near miss criteria were in vogue for some years, lack of uniformity was the hindrance. WHO criteria, 2009 [6] are unique in considering not only clinical but also laboratory and management-based criteria. Hence it incorporates both Mantel's [8] and Waterston's criteria [9]. So if one of the criteria fails to pick the case, the other makes it up, thus minimizing the chance of missing the case. 

A study by Jayarathnam et al. [10] represents near miss from a developed country, and results are as expected; preeclampsia, PPH, and sepsis are the major causes. Probably the study would have been more complete if the authors had commented about the maternal mortality ratio and near miss to maternal mortality ratio. In particular, the comparison will represent the improvement in the quality of care. In comparison, hemorrhage was the same, but near miss ratio is almost three times more in our study obviously differentiating the developing country (India) from developed country (Australia). Other countries like Nepal, Syria, African countries and Indonesia have shown similar trends in near miss incidences [11–15].

SAMM study from Brazil was in ICU setting only, while our study included ICU, high dependency unit, and labor room and hence represent all cases of near miss. Our study results were comparable to the studies of other developing countries, but these studies did not consider the WHO criteria [16].

The maternal near miss incidence ratio (MNMR) was 17.8/1000 live births in our hospital. Studies done in the developing countries show the same trend and vary from anywhere between 15–40/1000 live births [6, 17, 18]. The above studies have used various criteria for identification of the cases. A cross-sectional study done in Brazil using the Mantel's and Waterson's criteria showed a varying pickup rate of 62 and 86, respectively [19]. So some variation in the pickup rate from other studies might be there with the WHO criteria.

The maternal mortality ratio at our setup was 313/100000 live births. The Brazilian study showed a similar mortality rate of 260/100000 live births [18]. In other developing countries the maternal mortality ratios were 423/100000 live births and 324/100000 live births [11, 12]. Sepsis is on the rise due to epidemics of viral infections. Unlike our study hemorrhage is still the major cause in other developing countries. Hemorrhage was the second leading cause. There were 3 deaths, 2 following ruptured ectopic and one postpartum hemorrhage in 2011 and only one during 2012. The cases that were referred were in an already exsanguinated state. Though we have 24 h working blood bank with cell separator, hence component therapy, we were not able to revive these late referred cases. Important to note here is that there was not a single death due to hypertension in the year 2012. Postcesarean infections have contributed to mortality and have been a cause of worry.

The near miss to mortality ratio was 5.6 : 1, which means for every five to six life threatening conditions there was one maternal death. Higher ratios indicate better care. Syrian study showed a ratio of 60 : 1 and study done in Nepal showed a ratio of 7.2 : 1 [11, 12]. This ratio is similar to those of African country where the range is 1 : 5–12 [19]. This is a far cry from those reported in Western Europe. Their studies have reported a ratio of 117–223 : 1 [17]. If this ratio increases over a period of time, it reflects on the improvement achieved in obstetric care. So instead of a single estimation, yearly estimation may help us in improving the care provided.

 Ours is a tertiary referral center covering three other districts in and around Udupi, with most of the cases being referred in an already moribund state. The delays in referrals are a major cause of morbidity and mortality. Establishment of a tertiary care in each district is essential. Delayed diagnosis, inappropriate transfer, and inadequate utilization of resources might have been the cause for maternal morbidities and mortalities in our study. Along with increased awareness of one's own health, health education may go a long way in improving the quality of obstetric care. 

**Figure 1:**
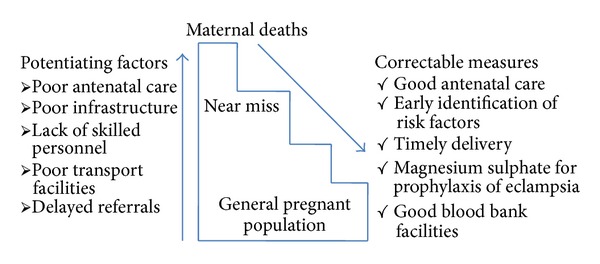
Diagram representing potentiating factors of maternal death and the correctable measures which need to be undertaken.

In Figure 1, right hand box represents the risk factors for morbidity and mortality, while the factors in the left hand box cause a stepwise decreasing trend in the path towards morbidity and mortality [20].

Limitation of the study is that as ICU facilities were available few potentially life threatening conditions before going on to near miss might have been selected. It is a single audit; data collection spanning over a few years would give a true picture of the improvement in obstetric care and also the long-term effects of SAMM. 

## 5. Conclusion

 Hemorrhage and hypertensive disorders are the leading causes of near miss events. New-onset viral infections have emerged as the leading cause of maternal mortality. The trend of near miss events and maternal deaths is the same in the two years. As near miss analysis indicates quality of health care, it is worth presenting in national indices. 

## Figures and Tables

**Table 1:** Characteristics of near miss cases and maternal deaths.

Characteristics	Near miss, *n* = 131, (%)		Total	Maternal deaths, *n* = 23, (%)		Total
	2011	2012		2011	2012	
Age (years)	27.3 ± 4.75 (SD)	26.7 ± 4.6	27.0 ± 4.7	27.8 ± 4.8 (SD)	27.6 ± (4.4)	27 ± (4.5)
Parity						
Primipara	37 (54.4)	37 (58.7)	74 (56.4)	4 (36.3)	7 (58.3)	11 (47.8)
Multipara	31 (45.6)	26 (41.3)	57 (43.6)	7 (63.6)	5 (41.6)	12 (52.2)
Gestational age (weeks)						
1–12	7 (10.3)	10 (15.9)	17 (12.9)	3 (27.3)	1 (8.3)	4 (17.4)
13–28	3 (4.4)	3 (4.8)	6 (4.5)	2 (18.1)	0	2 (8.7)
>28	43 (63.2)	32 (50.8)	*75 (57.2) *	3 (27.3)	3 (25)	6 (26.1)
Postnatal	15 (22.1)	18 (28.6)	*33 (25.1) *	3 (27.3)	8 (66.6)	*11 (47.8) *
Causes						
Hemorrhage	26 (38.2)	32 (50.8)	*58 (44.2) *	3 (27.3)	1 (8.3)	4 (17.4)
Hypertension	24 (35.3)	07 (11.1)	31 (23.6)	1 (9.1)	0	1 (4.3)
Sepsis	9 (13.2)	12 (19)	21 (16)	5 (45.5)	7 (58.3)	12 (52.2)
Cardiac	2 (2.9)	4 (6.3)	6 (4.5)	2 (18.1)	2 (16.6)	4 (17.4)
Indirect	7 (10.3)	8 (12.7)	15 (11.4)	0	2 (16.6)	2 (8.7)

**Table 2:** Comparison of near miss events and primary causes of maternal deaths.

Diagnosis	Near miss		Near miss/1000 live births	Mortality		Mortality index %
	2011	2012		2011	2012	
Hypertensive disorders of pregnancy	**24 **	**7**	***4.2***	**1**	0	***3.1***
Severe preeclampsia	8	1		0		
Eclampsia	10	4		1		
HELLP syndrome	6	2		0		
Severe haemorrhage	**26**	**32**	***7.9***	**3**	**1**	***6.5***
Early pregnancy				2		
Ectopic pregnancy	2	8		0		
Abortion	1	1				
Late pregnancy				0		
Abruption	3			1		
PPH	20	23				
Sepsis	**9**	**12**	***2.9***	**5**	**7**	***36.3***
H1N1	0			3		
Others	9			2		
Cardiac	**2**	**4**	***0.8***	**2**	**2**	***40***
Indirect	**7**	**8**	***2***	**0**	**2**	***11.8***

Total	**68**	**63**	***17.9***	**11**	**12**	***14.9***

## Abbreviations

MD: Maternal death
MNMR: Maternal near miss incidence ratio
MI: Mortality index
MMR: Maternal mortality ratio
SAMM: Severe acute maternal morbidity.
